# Correspondence

**Published:** 1873-09

**Authors:** C. M. Wright

**Affiliations:** Basel, Switzerland


### CORRESPONDENCE.

BASEL, SWITZERLAND, FEB. 12th, 1873

DR. H. A. SMITH:—I received the circular programme of the dear old Mississippi Valley Dental Association to-day, to which your name is attached, and will take the liberty of sending through you, *Greeting!* to the association. You have kindly read for me on other occasions, essays that have been "pitched into" by brother dentists. As time rolls on I see no reason for changing the views expressed in those essays, though at all times open to conviction.

Since the last meeting of our Association, I have changed entirely my field of labor. There is some difference in the class and quality of the teeth here presented, but that difference is not material, and probably in certain sections of America, the same quality presents. As a rule the teeth are softer, and caries (of the light colored and soft variety) predominates. In a majority of children's teeth, the caries attacks the teeth on their approximal surfaces and near the margin of the gum, so that particularly careful examinations are necessary. The majority of our Swiss patients take excellent care of their teeth, and yet salivary calculus of the light brown, the hard and dark and the

"green stain" varieties abound. All along the valley of the Rhine, the teeth are of inferior quality and also subject to calculus.

The better classes do not eat the brown bread so highly recommended by Dr. John Allen, and all classes indulge in the light wines of the neighborhood; most of the wines are of a sharp acid taste to an American. Typhus fever is a prevalent disease here, and if you will allow a side remark, the latest *cure* recommended by our most scientific physicians, is *the bath*, the the tepid sponge bath in some cases, and in others, when the fever is high, the cold bath; some physicians recommend the bath every two hours. The treatment is quite popular in Switzerland, France and Germany; neuralgia and rheumatism are also common diseases. These are all of course detrimental to the teeth. I think it is noticeable, that the teeth of the *Swiss* are not, on the whole, as good in form and quality as the teeth of Americans; irregularities of arrangement, and malformed enamel and irregular dentition are more common than in the neighborhood of Cincinnati. Where did the notion arise, that "Europeans have better teeth than Americans?" If *civilization* is to be charged with the destruction of the human teeth, why should not these old civilized countries expose the defects.

I am very much pleased with the practical programme of discussions your executive committee have offered, and I heartily wish some of your members may, with no fear of the meaning shrug of the shoulders of some of the *advanced*, boldly criticise the popular method of *mouth architecture*—I mean the advanced notion of restoring every PARTICLE of form of the diseased tooth with gold—and thereby in many cases disfiguring the mouth. I mildly protested against this in an essay, a year or two ago, and was taken in hand by Dr. Driscoll; I did not see or feel the *force* of his arguments and did not reply. I would like to say it again: *I think it is damnable to blast the expression God has given to the human face, because, forsooth, we can train our fingers to apply a rubber dam, and make pellets of gold or mats of gold stick together.* I insist that it requires more skill, and is infinitely less presumptive and in better taste to hide our art, and improve, or restore expression to the face.

An English writer once described America, as the "place where boots are imperfectly polished;" we have got over that, but any foreign or native writer, might to-day with justice describe Americans, as "people who have gold in their front teeth." The people here have better taste, I suppose *Dr. Advance* would say "they are not *educated up* to the *standard* yet. No, and they shall not be by me, for I invariably teach that it is a misfortune to have to exhibit gold in the teeth. In regard to Dr. Arthur's method, I am not bold enough to practice it fully, but am working cautiously in the direction of his advice, and watching results. It is a very plausihle theory, and I shall adopt it as soon as I can base it on my own practice.

In mechanical dentistry, *plain teeth* do better here than at home, which also suits my *theory*, and I am glad to know that another member of your society attributes the decline in art in the mechanical department to the introduction and use of gum and block teeth,—and it was a sorry day for the dental profession, when they declined to encourage Dr. McClelland's Rose-Pearl—a sorry day I mean, because Rose-Pearl offered a cheap, beautiful and durable medium, through which the profession might *restore* the lost *art*, and advance it to a place by the side of the painter and sculptor. Dr. Palmer and some others are not willing to give up the artistic capabilities of the mechanical department so called, as his specimens and diagrams exhibited last year proved, but if Rose-Pearl had been adopted, we would have the *great* "continuous gum work," wonderfully assisted by the beautiful handmaid "Rose-Pearl," and the mechanical dentist would no longer be recognized as a member of our profession ; in his place would stand the "dental and facial artist"—a gentleman who would be born to fine white linen shirts, and dainty cuff-buttons, and who would not be ashamed to say, "I am a dental artist, madam,"—such language as "*puttin' up a job on rubber*," would then never be heard outside of the village blacksmith shop. Let us pray for a revival, brethren. Let us go ourselves into our laboratories and not apply ourselves, as we have, to disfiguring front teeth with gold, and may be after a while we may have an era of *good taste* among dentists.

With the keenest feelings of sorrow that I can't be with you in the body, I remain very truly yours. C. M. Wright.

